# Sequence of focal choroidal excavation types in a patient with bilateral central serous chorioretinopathy

**DOI:** 10.5935/0004-2749.20210063

**Published:** 2021

**Authors:** Luiz H. Lima, Luiz Guilherme Marchesi Mello, Júlia Polido, Laurentino Biccas Neto, Fábio Petersen Saraiva, Thiago Cabral

**Affiliations:** 1 Department of Ophthalmology, Escola Paulista de Medicina, Universidade Federal de São Paulo, São Paulo, SP, Brazil.; 2 Department of Specialized Medicine, Universidade Federal do Espírito Santo, Vitória, ES, Brazil.; 3 Vision Center Unit, Ophthalmology, Hospital Universitário Cassiano Antonio Moraes, Universidade Federal do Espírito Santo, Vitória, ES, Brazil.; 4 Ocular Oftalmologia Clinic, Espírito Santo, Brazil.

**Keywords:** Central serous chorioretinopathy, Choroid, Focal choroidal excavation, Tomography, optical coherence, Retina, Coriorretinopatia serosa central, Coroide, Escavação focal de coroide, Tomografia de coerência óptica, Retina

## Abstract

A 39-year-old policeman complained of decreased bilateral central vision over the last two years. On examination, visual acuity was 20/40 and 20/400 in the right (OD) and left eye (OS), respectively, and fundoscopy revealed a bilateral hypopigmented macular lesion. Fluorescein and indocyanine green angiography demonstrated leakage and hyperintense spots, respectively, within the macular areas. At baseline, optical coherence tomography showed subretinal fluid in the OD and a conforming focal choroidal excavation in the OS. Focal choroidal excavation converted from conforming to nonconforming type at 4-month follow-up and then reversed to conforming type at 12-month follow-up, and was associated with incomplete retinal pigment epithelium and outer retina atrophy over the area of excavation. Pachyvessels were also evidenced in the choroid, without neovascularization. We report for the first time a case of focal choroidal excavation that progressed from conforming to nonconforming type and then reverted to its primary configuration (conforming type) in a patient with concurrent bilateral central serous chorioretinopathy.

## Abstract (pt)

Um policial de 39 anos se queixava de diminuição bilateral da visão central nos últimos 2 anos. A acuidade visual era 20/40 e 20/400 no olho direito (OD) e esquerdo (OE) e a fundoscopia revelou lesão macular hipopigmentada bilateral. A angiografia fluoresceínica e com indocianina verde revelaram, respectivamente, vazamento do corante e áreas hiperintensas nas regiões maculares. A tomografia de coerência óptica evi denciou fluido sub-retiniano no OD e escavação focal de coroide do tipo conformacional no OE. Após 4 meses, a escavação focal de coroide mudou de conformacional para não conformacional e, aos 12 meses, reverteu para conformacional associado a atrofia incompleta do epitélio pigmentar da retina e da retina externa na região da escavação. Também foi evidenciado paquicoroide, sem neovascularização. Relatamos pela primeira vez uma escavação focal de coroide que evoluiu de conformacional para não conformacional e, em seguida, retornou à configuração primária (conformacional) em um paciente com coriorretinopatia serosa central bilateral.

## INTRODUCTION

Focal choroidal excavation (FCE) was first described by Jampol in 2006 and corresponds to a localized area of retinal depression with an underlying choroidal thinning^(1)^. On fundoscopy, FCE shows as a small lesion with pigmentary changes in the posterior pole. Its etiology is poorly understood, with congenital posterior segment malformation and previous choroidal scarring suggested as possible explanations^(2,3)^.

There are some reports of FCE associated with central serous chorioretinopathy (CSC), and both diseases share similar choroidal findings on fluorescein (FA) and indocyanine green angiography (ICGA)^(4-6)^. The choroidal hyperpermeability and hypofluorescent spots found in CSC may also be observed in FCE eyes with CSC. These findings may be related to a choroidal hemodynamic impairment^(7,8)^. Besides CSC, other conditions have been associated with FCE, including pachychoroid disease^(9)^ and multiple evanescent white dot syndrome^(10)^. Other important clinical manifestations of the pachychoroid spectrum disease, besides FCE and CSC, are pachychoroid pigment epitheliopathy, pachychoroid neovasculopathy, polypoidal choroidal vasculopathy/aneurysmal type 1 neovascularization, and peripapillary pachychoroid syndrome^(11)^. In addition to FA and ICGA, other imaging features allow better characterization of the FCE: i) optical coherence tomography (OCT) may reveal high hyperreflective choroidal thinning in the area of the FCE, a separation or attachment between photoreceptors and the retinal pigment epithelium (RPE), and absence of abnormalities of the underlying sclera^(11)^; ii) swept-source, enhanced depth imaging, and three-dimensional OCT can improve choroid and sclera analysis^(2,4,12)^; iii) OCT angiography may demonstrate alteration of the deep capillary and choriocapillaris plexus^(13)^.

Based on OCT findings, FCE is classified into two types: conforming (without separation between photoreceptor tips and RPE) and nonconforming (with separation between photoreceptor tips and RPE)^(2)^. Some studies report FCE progression from the conforming to nonconforming type and vice-versa but none describe FCE reversion to its initial configuration^(3-5)^. Furthermore, OCT is also helpful in measuring the FCE (horizontal - greatest linear dimension - and vertical lengths)^(12,14)^ and to classify it according to its morphology (cone-shaped, bowl-shaped, or mixed type)^(8)^. We report a unique case of bilateral CSC in which a large bowl-shaped FCE progressed from the conforming to the nonconforming type and then reverted to its primary arrangement (conforming).

## CASE REPORT

**Figure 1:**
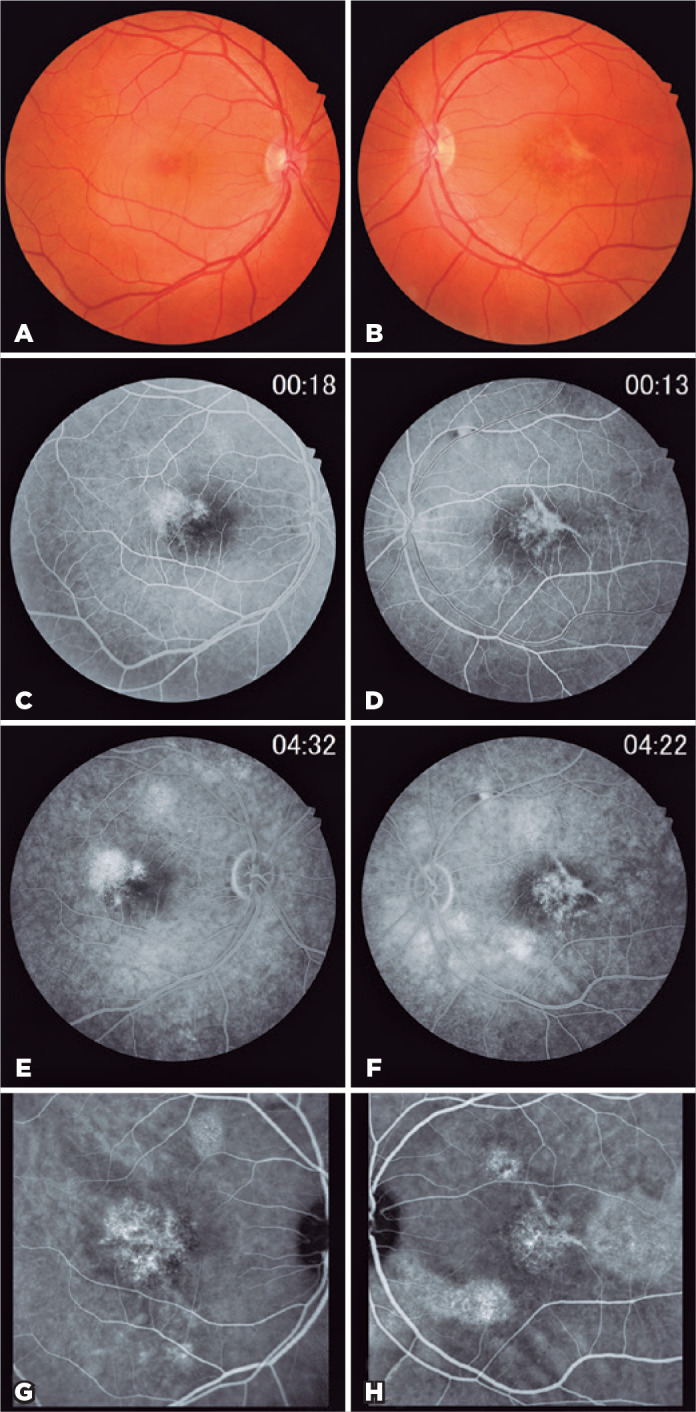
A and B) Color fundus photograph of right and left eyes demonstrated a hypopigmented lesion within the macula. C-F) Fluorescein angiography showed a small leakage within both macular areas. G and H) Indocyanine green angiography of both eyes demonstrated choroidal hyperpermeability and hyperfluorescent spots within the macula.

**Figure 2:**
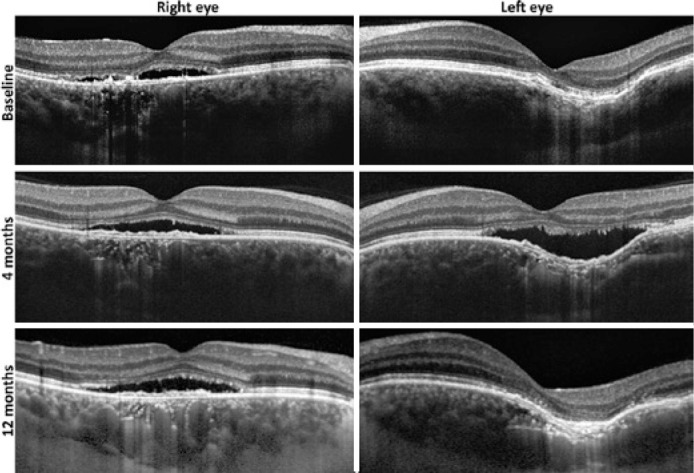
Spectral-domain optical computed tomography (OCT) demonstrated increased choroidal thickening in both eyes, persistent subretinal fluid in the right eye during follow-up (Baseline, 4 and 12 months), and left-eye focal choroidal excavation (FCE) conversion from conforming (baseline) to nonconforming (4 months) with a greatest linear dimension of 2.738 _µ_m. Total resolution of subretinal fluid and reversion of FCE type from nonconforming to conforming were observed in the left eye at 12-month follow-up, associated with incomplete RPE and outer retina atrophy (iRORA) over the area of excavation.

A 39-year-old policeman complained of decreased bilateral central vision over the previous two years. He reported that the poor vision began a few weeks after returning to work in external activities in rotating shifts. His previous ocular and systemic history were unremarkable. On examination, the best-corrected visual acuity (BCVA) was 20/40 and 20/400 in the right (OD) left eye (OS), respectively. Slit-lamp biomicroscopy of the anterior segment and intraocular pressure were unremarkable. Fundoscopy showed a hypopigmented lesion within the macular areas (Figure 1A-B). FA revealed a macular window defect corresponding to RPE atrophy and small leakage within both macula (Figure 1C-F). ICGA demonstrated choroidal hyperpermeability and hyperfluorescent spots in both macula (Figure 1G-H). OCT showed subretinal fluid in the OD, a conforming bowl-shaped FCE in the OS (Figure 2 - baseline), and increased bilateral choroidal thickness. The diagnosis of bilateral CSC was based on the medical history, serous retinal detachment, and angiographic leakage.

At 4-month follow-up, BCVA was 20/50 in the OD and 20/400 in the OS. OCT showed subretinal fluid and conversion from conforming to nonconforming FCE type in the OS (Figure 2: 4 months). Oral spironolactone (25 mg twice daily for two months) was initiated. After one year, BCVA was 20/50 in the OD and 20/200 in the OS, there was a complete resolution of subretinal fluid, and the FCE had reverted from the nonconforming to conforming type, associated with incomplete RPE and outer retina atrophy (iRORA) over the excavation. Pachyvessels were evidenced in the choroid during follow-up (Figure 2), with no evidence of neovascularization.

## DISCUSSION

The association of FCE with CSC has been previously reported, with prevalence ranging from 2.8% to 24.4%^(4,6)^. One of the hypotheses of the pathogenesis of FCE in CSC eyes is an abnormal tractional force driven by unknown choroidal scarring that may cause contractive forces on the RPE in a thickened choroid in eyes with acute CSC^(2,3)^. FCE and CSC may present common clinical features since choroidal vascular hyperpermeability with subsequently subretinal fluid accumulation and punctate hyperfluorescent spots on ICGA have been reported in both conditions^(7,8)^. Similarly to CSC, increased choroidal thickness has been noted in FCE, although it is unknown whether it is an intrinsic feature of FCE eyes or whether it occurs only in eyes with FCE and CSC^(3,4)^. In our case, the patient presented bilateral CSC and unilateral FCE. The FCE was located within the area of fluorescein leakage and choroidal hyperpermeability, as previously described^(3,4)^.

A review of the literature found two previous cases that developed CSC after the diagnosis of FCE and pachychoroid disease. The authors of one study hypothesized that FCE had exacerbated the choroidal ischemia with subsequent damage to the RPE / Bruch’s membrane complex, predisposing the development of CSC^(9)^. However, it is uncertain whether the presence of CSC was a coincidence, or if it was related to a defective choroidal structure in the FCE.

According to the attachment between the photoreceptor tips and the RPE, there are two types of FCE. In conforming FCE, the photoreceptor tips and the RPE are attached and have a normal structure. In nonconforming FCE, the photoreceptor tips are detached from the underlying RPE and a hyporreflective space is observed between them, probably representing subretinal fluid^(3)^. In our case, FCE presented as the conforming type, but became nonconforming after 4 months, and then converted to its initial configuration (conforming) at the 12-month follow-up. Although Margolis et al.^(2)^ hypothesized that conforming FCE progresses to nonconforming lesions when stress on the outer retina leads to the photoreceptor’s tips detachment from the RPE, conforming FCE may spontaneously convert to nonconforming. In this setting, serous or hemorrhagic retinal elevation resulting from active CSC or choroidal neovascularization (CNV) is observed^(2,3)^. Lee et al. reported two eyes with nonconforming FCE and active CNV that reverted to conforming FCE after CNV resolution^(3)^. This FCE reversion was probably due to subretinal fluid absorption following inactivation of CNV. In our case, the change from conforming to nonconforming type was possibly related to retinal elevation due to subretinal fluid accumulation from CSC instead of the progression of the excavation itself. The further reversion (nonconforming to conforming) at 12-month follow-up may be linked to the resolution of serous retinal detachment. Probably, the transformation from nonconforming to conforming type occurred following subretinal fluid absorption and, consequently, outer retina breakdown into the excavation. OCT angiography would provide additional data, such as analysis of the retinal and choroidal vascular plexus.^(13)^ However, in this case, the performed multimodal imaging analysis (FA, OCT, and ICGA) allowed the characterization of pachychoroid spectrum disease-associated abnormalities.

We report a case of bilateral CSC associated with unilateral FCE that progressed from the conforming to the nonconforming type and then reverted to its primary OCT arrangement. There is no other reported case of FCE associated with CSC that shows this sequence of FCE types. Larger series of patients with both FCE and CSC and longer follow-up are needed to obtain other similar clinical cases and to establish whether this sequence of FCE types is related to the retinal and choroidal morphological changes that occur during the progression of CSC disease.
